# Advances in assessment and cognitive neurorehabilitation of HIV-related neurocognitive impairment

**DOI:** 10.1093/braincomms/fcae399

**Published:** 2024-12-26

**Authors:** Elia L Fischer, Alexis Renaud, Petr Grivaz, Giovanni Di Liberto, Philippe Ryvlin, Matthias Cavassini, Renaud A Du Pasquier, Arseny A Sokolov

**Affiliations:** NeuroScape@NeuroTech Lab, Service Universitaire de Neuroréhabilitation (SUN), Département des Neurosciences Cliniques, Centre Hospitalier Universitaire Vaudois (CHUV), Institution de Lavigny, University of Lausanne, 1011 Lausanne, Switzerland; Department of Neurology, Inselspital, Bern University Hospital, University of Bern, 3010 Bern, Switzerland; NeuroScape@NeuroTech Lab, Service Universitaire de Neuroréhabilitation (SUN), Département des Neurosciences Cliniques, Centre Hospitalier Universitaire Vaudois (CHUV), Institution de Lavigny, University of Lausanne, 1011 Lausanne, Switzerland; NeuroScape@NeuroTech Lab, Service Universitaire de Neuroréhabilitation (SUN), Département des Neurosciences Cliniques, Centre Hospitalier Universitaire Vaudois (CHUV), Institution de Lavigny, University of Lausanne, 1011 Lausanne, Switzerland; Service of Neurology, Department of Clinical Neurosciences, Lausanne University Hospital, University of Lausanne, 1011 Lausanne, Switzerland; Département de Pathologie et Immunologie, Université de Genève, 1206 Geneva, Switzerland; Service of Neurology, Department of Clinical Neurosciences, Lausanne University Hospital, University of Lausanne, 1011 Lausanne, Switzerland; Division of Infectious Diseases, Lausanne University Hospital, University of Lausanne, 1011 Lausanne, Switzerland; Service of Neurology, Department of Clinical Neurosciences, Lausanne University Hospital, University of Lausanne, 1011 Lausanne, Switzerland; NeuroScape@NeuroTech Lab, Service Universitaire de Neuroréhabilitation (SUN), Département des Neurosciences Cliniques, Centre Hospitalier Universitaire Vaudois (CHUV), Institution de Lavigny, University of Lausanne, 1011 Lausanne, Switzerland

**Keywords:** human immunodeficiency virus, HIV-associated neurocognitive disorder, neurotechnology, serious video games, cognitive neurorehabilitation

## Abstract

Neurocognitive impairment (NCI) is present in around 40% of people with HIV and substantially affects everyday life, adherence to combined antiretroviral therapy (cART) and overall life expectancy. Suboptimal therapy regimen, opportunistic infections, substance abuse and highly prevalent psychiatric co-morbidities contribute to NCI in people with HIV. In this review, we highlight the need for efficacious treatment of HIV-related NCI through pharmacological approaches and cognitive neurorehabilitation, discussing recent randomized controlled trials in this domain. We also discuss the benefits of a thorough and interdisciplinary diagnostic work-up between specialists in neurology, psychiatry, neuropsychology and infectious diseases, helping to disentangle the various factors contributing to cognitive complaints and deficits in people with HIV. While the advent of cART has contributed to slowing the progression of cognitive deficits in people with HIV and reducing the prevalence of HIV-associated dementia, NCI persists at a significant rate. Adjuvant stimulating or neuroprotective pharmacological agents have shown some potential benefits. Despite promising outcomes, studies on cognitive neurorehabilitation of HIV-related NCI remain sparse and limited in terms of methodological aspects. The access to cognitive neurorehabilitation is also restricted, in particular at the global scale. Novel technology bears a significant potential for restoring cognitive function in people with HIV, affording high degrees of standardization and personalization, along with opportunities for telerehabilitation. Entertaining serious video game environments with immersive graphics can further promote patient motivation, training adherence and impact on everyday life, as indicated by a growing body of evidence, including in seropositive children and older individuals in Africa. Upon validation of technology-assisted cognitive neurorehabilitation for HIV-related NCI in large-scale randomized controlled trials with state-of-the-art methodology, these approaches will promote socio-professional reintegration and quality of life of people with HIV.

## Teaser

Neurocognitive impairment affects 40% of HIV patients, disrupting daily life and treatment adherence. Different specialists are vital for diagnosis. Regarding treatment, studies on cognitive neurorehabilitation yielded promising results but more research is needed to ultimately improve life in affected patients.

## Graphical Abstract

**Graphical Abstract:**
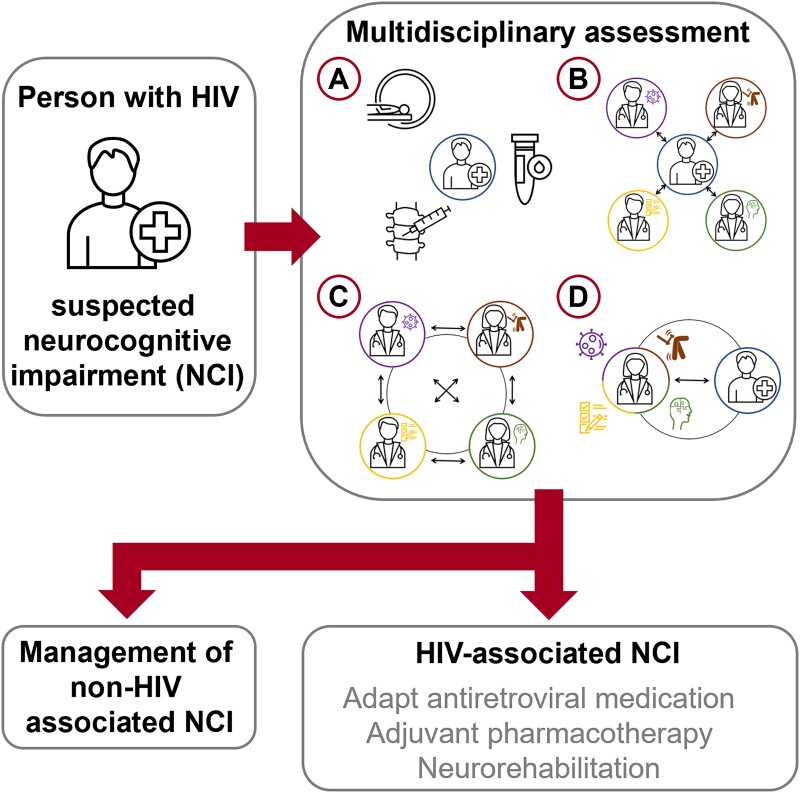

## Introduction

Human immunodeficiency virus (HIV) affects ∼38 million people worldwide and around 1.7 million individuals are infected each year.^1^ The advent of combined antiretroviral therapy (cART) has been essential in addressing the primary issue of survival.^2^ Despite this progress, patients and care providers continue facing significant challenges related to the HIV infection, in particular in the social and cognitive domains.^3^ Cognitive symptoms are observed in 20–60% of people with HIV^4,5^ and may not only interfere with everyday function and quality of life^6,7^ but also medical adherence^8^ and mortality.^9,10^ The most affected cognitive domains are processing speed, attention, working memory and cognitive flexibility.^4,11^

To classify the severity of the subjective and objective cognitive impairment, Antinori *et al*.^12^ proposed the Frascati criteria and divided HIV-associated neurocognitive disorders into three categories: asymptomatic neurocognitive impairment (NCI), mild neurocognitive disorder and HIV-associated dementia. In the past years, this classification has been increasingly challenged.^4,13-15^ One reason is that a score of lower than 1 SD below the mean in at least two out of seven domains is enough to qualify for asymptomatic NCI,^12^ even though other factors such as cultural differences or co-morbidities may account for these results.^13^ The resulting high rate of false positives is considered problematic and stigmatizing for affected people with HIV; consequently, the clinical relevance of asymptomatic NCI has been questioned.^13,16,17^ As challenges remain in defining cognitive deficits in people with HIV, alternative classifications have been proposed^18^ and a global term of NCI will be used in this review. As NCI among people with HIV still poses major diagnostic and therapeutic challenges, the principal aim of this review is to discuss current directions for pharmacological and non-pharmacological treatment of HIV-related NCI, following interdisciplinary determination of the aetiology and exclusion of differential diagnoses.

### Interdisciplinary assessment and management of cognitive deficits in people with HIV

**Figure 1:**
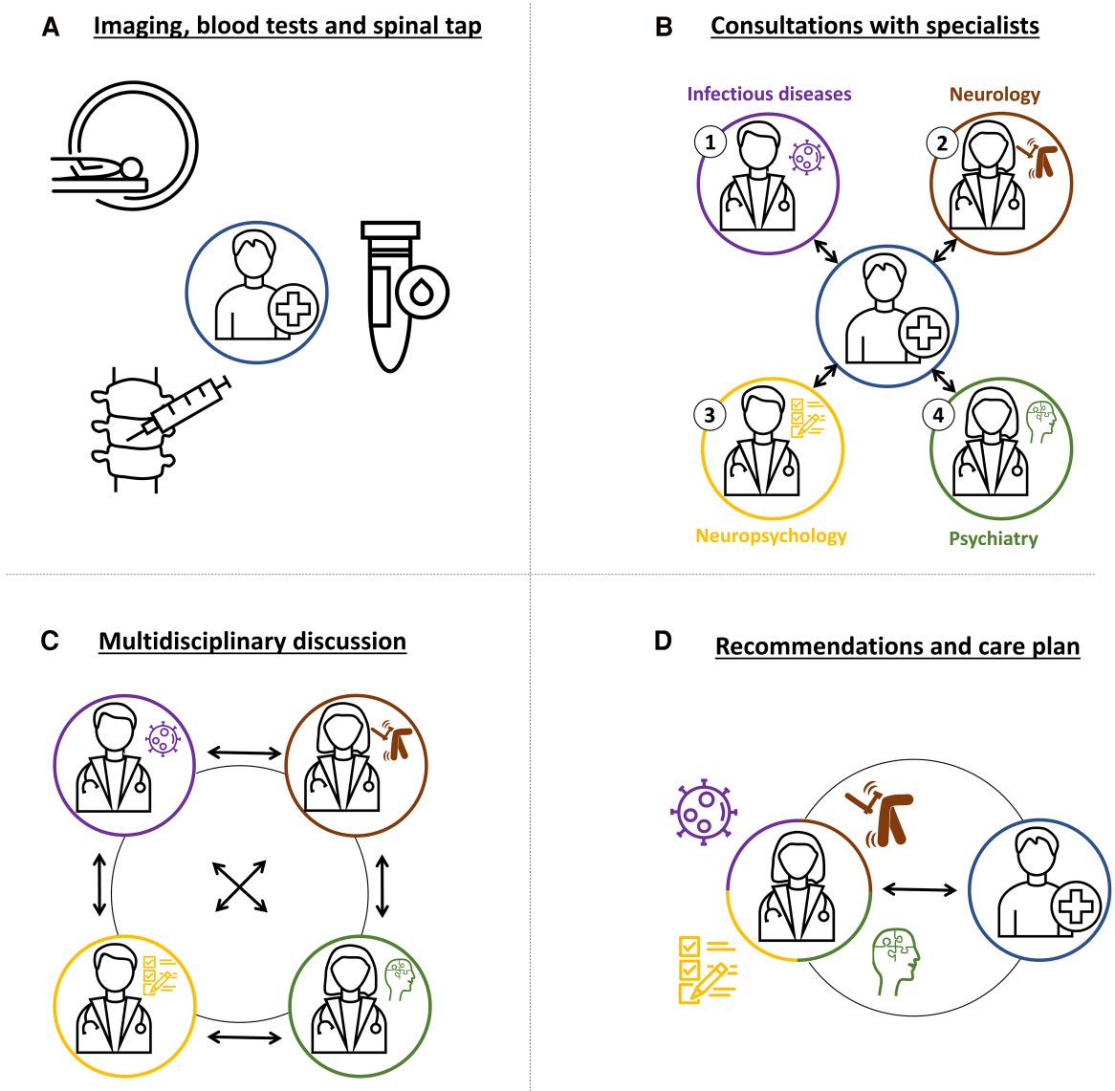
**Proposed structured and interdisciplinary work-up of cognitive complaints in people with HIV.** During a 1-day interdisciplinary outpatient work-up, (**A**) patients undergo brain MRI, blood testing including cART monitoring and HIV viral load, and a lumbar puncture including HIV-RNA levels. (**B**) During the clinic, patients are seen by (1) the infectious diseases specialist, (2) neurologist, (3) neuropsychologist and (4) psychiatrist. (**C**) The evaluations are concluded by an interdisciplinary discussion between the four specialists, reviewing the clinical, imaging and laboratory findings, defining the aetiology and suggesting a management plan that may involve pharmacological adaptations, psychotherapy and neurorehabilitation. (**D**) The findings, diagnostic considerations and suggestions are discussed with the patient.

Identifying the precise aetiology of the cognitive deficits in people with HIV and their management is a complex endeavour given the impact of other highly prevalent factors contributing to cognitive dysfunction, such as psychiatric co-morbidities, substance use and other conditions.^18,19^ In order to account for the multifactorial origin of NCI in people with HIV, a recent expert consensus statement proposed to differentiate HIV-associated brain injury (HABI) itself from other causes of brain damage.^13^ To better differentiate the contribution of HABI as opposed to other factors, interdisciplinary approaches and holistic frameworks appear indispensable.^13,20-22^ Especially in advanced NCI among people with HIV, finding the link between subjective complaints, objective deficits and the underlying aetiology requires interdisciplinary teams with a patient-centred approach. Therefore, a structured work-up involving experts from infectious diseases, neurology, neuropsychology, psychiatry and neurorehabilitation is recommended in diagnosing HIV-associated NCI (Fig. 1),^21^ following screening and pre-selection of patients requiring such an assessment. The widely used screening tests Mini Mental State Examination and the Montreal Cognitive Assessment demonstrated an insufficient sensitivity for detecting NCI in people with HIV.^23,24^ In accordance with a recent expert consensus^13^ and the European AIDS Clinical Society guidelines,^21^ it may be more advisable to shift the focus away from objective cognitive function and towards cognitive complaints raised by people with HIV. Consequently, we recommend a short screening questionnaire with three questions on memory, executive and attentional functions.^25^ In addition, neuropsychological examination may be used for pre-selection, to confirm the presence of objective cognitive deficits requiring further investigation.

During the proposed work-up, an infectious disease consultation assesses the patients’ lifestyle, cognitive complaints and adherence to cART. Persistent viral load in the plasma or therapy regimen with known harmful cognitive side effects^26^ should prompt a change in cART. Switching to medications with a more favourable effect on cognition has been shown to improve subjective and objective cognitive function.^27,28^ A neurological assessment and a brain MRI (if available) should be conducted to screen for other central nervous system (CNS) pathologies or causes of cognitive decline. A CNS viral escape syndrome as a cause of progressive CNS damage due to HABI^13^ should be ruled out by a lumbar puncture allowing to measure HIV-RNA levels in the cerebrospinal fluid (CSF). This syndrome may occur in some patients in which the virus continues to replicate in the CSF despite an undetectable viral load in the plasma and effective cART treatment.^29,30^ As people with HIV have an overall higher prevalence and earlier onset of Alzheimer’s disease,^31^ measuring beta-amyloid and phosphorylated-tau in the CSF of patients over 55 years of age may also be helpful depending on the cognitive and radiological profile. A further essential component of the work-up is a comprehensive neuropsychological assessment,^15,32,33^ including tests on the seven most important functional domains (Table 1).^4,12^

**Table 1:** Suggested neuropsychological tests for the work-up of HIV-related NCI

Ability domain	Neuropsychological test
Verbal/language	Hopkins Verbal Learning Test—Revised Phonemic fluency Semantic fluency
Attention/working memory	Forward Span of the Wechsler Adult Intelligence Scale 4th edition Backward Span of the Wechsler Adult Intelligence Scale 4th edition
Abstraction/executive	Trail Making Test B or Colour Trail Test 2 Stroop Test Wisconsin Card Sorting Test Five-point test
Memory (learning and recall)	Hopkins Verbal Learning Test-Revised Rey–Osterreith Complex Figure Test
Processing speed	Digit Symbol test of the Wechsler Adult Intelligence Scale 4th edition or Symbol Digit Modalities Test Trail Making Test A or Colour Trail Test 1
Sensory-perceptual	Grooved Pegboard Test
Motor skills	Finger-Tapping Test Grooved Pegboard Test

The pool of neuropsychological tests usually employed in the Lausanne Neuro-HIV platform, according to international recommendations.^12,15,32,33^ Of note, the test selection is adapted to patient’s complaints, age and educational level, including alternative or more in-depth tests.

As proposed in the HABI framework, the neuropsychological findings should be interpreted within the clinical and socio-cultural context, in order to determine their true impact on everyday life and the underlying aetiologies.^13^ To this end, it may be helpful to include patient-reported outcome measures such as the Lawton’s Instrumental Activities of Daily Living to assess activities necessary for independent living^34^ or the Medical Outcomes Survey-HIV to measure health-related quality of life in people with HIV.^35^

The high prevalence of affective and psychotic disorders in people with HIV and their potential impact on cognitive function mandate a thorough psychiatric evaluation.^4^ Finally, an interdisciplinary board determines the most likely aetiology of the cognitive impairment and suggests treatment options for HABI and treatable co-morbidities contributing to NCI. These suggestions may include changes in cART regimen towards greater CNS penetration or lower CNS toxicity, introduction or adaptation of pharmacological treatment for psychiatric co-morbidities, the recommendation of psychotherapy or cognitive neurorehabilitation.

## Materials and methods

For the following narrative review sections focusing on neurorehabilitation and pharmacological treatment options for NCI, we conducted an online PubMed and Scopus research until May 2024. Our PubMed search included the MeSH-terms ‘HIV’, ‘AIDS Dementia Complex’, ‘Drug Therapy’, ‘Neurorehabilitation’ and keywords such as ‘brain function’, ‘cognitive’, ‘cognition’, ‘pharmacotherapy’, ‘neurorehabilitation’, ‘rehabilitation’, ‘improvement’, ‘training’, ‘stimulation’, ‘People living with HIV’, ‘PLWH’, ‘Human Immunodeficiency Virus’ and ‘HIV’. Our Scopus search included the terms HIV and cogn* together with either neurorehabil*, rehab* or pharm* in the titles, abstracts or keywords of listed publications.

### Pharmacological treatment of cognitive deficits in people with HIV

cART has been reported to slow down cognitive decline and to diminish HIV viral load in the CSF.^36,37^ Effective cART has also substantially reduced the prevalence of HIV-associated dementia.^11^ The CNS penetration-effectiveness (CPE) ranking system was developed to classify different cART regimens with respect to their ability to enter the CNS through the blood–brain barrier.^38^ An elevated CPE score means a greater capacity of the drug to penetrate the blood–brain barrier and thus the likelihood to affect the HIV-related mechanisms involved in cognitive impairment. An observational study on 94 patients reported that receiving a lower CPE cART regimen over 2 years was associated with cognitive decline.^39^ Therefore, treating patients with high CPE cART seemed a promising avenue. However, the scientific evidence in terms of clinical or cognitive benefits has remained controversial. A randomized controlled trial (RCT) comparing 16 weeks of higher CPE with lower CPE cART in 49 people with HIV with NCI did not find between-group differences in neuropsychological outcomes.^40^ Other data suggested that a higher CPE cART might actually result in poorer performance on neuropsychological tests^41^ or even in a higher incidence of HIV-associated dementia.^42^ These findings were interpreted as potential neurotoxic effects of higher CPE cART.^43^ The controversial findings preclude reliable conclusions on the efficacy of higher CPE cART in NCI due to HIV. Cognitive performance was not associated with the cumulative or cross-sectional CPE score of a large, well-characterized cohort in Switzerland.^44^ Furthermore, a recent RCT failed to show any benefit on the cognitive performance of people with HIV by adding one or two molecules (maraviroc ± dolutegravir) to an effective regimen.^45^ This is in line with other recent evidence that intensifying the antiretroviral therapy may not yield any additional benefit on cognitive function.^46^

Adjuvant therapies such as psychostimulant agents have also been studied in people with HIV. However, the nine reviewed RCTs (Table 2) did not provide evidence convincing enough to favour the recommendation of such an agent.

**Table 2:** A summary of research on pharmacological interventions for cognitive neurorehabilitation in NCI among people living with HIV

First author	Year	Study design	Intervention	Control	Population	Findings
Sacktor^47^	2000	Randomized, double-blind, placebo-controlled trial	Selegiline (MAO-Inhibitor), transdermal application of 1.0 mg/cm × 15 cm^2^ over 10 weeks (*n* = 9)	Transdermal placebo patch over 10 weeks (*n* = 5)	15 HIV patients	Significant improvement in the Rey Auditory Verbal Learning Delayed Recall and the Grooved Pegboard Test for the dominant hand
Hinkin^48^	2001	Single-blind, placebo-controlled crossover trial	Methylphenidate (phenylethylamine), oral application of 10 mg for 3 days (before or after placebo with in-between washout period of 3 days) (*n* = 16)	Oral placebo (before or after methylphenidate with in-between washout period of 3 days) (*n* = 16)	16 HIV patients	Improvement of choice reaction time and dual-task reaction time in participants with initially low choice reaction time
Letendre^49^	2006	Single-arm, open-label pilot study	Lithium, oral application beginning at 300 mg/d to maintenance concentration dose between 0.4 and 0.8 mEq/L (600 mg to 1200 mg/d) over 12 weeks (*n* = 8)	None	8 HIV patients	Improvement in a composite neuropsychological score (global deficit score) of seven cognitive domains
Schifitto^50^	2007	Randomized, placebo-controlled, three-arm study	Selegiline (MAO inhibitor), transdermal application of 3 (*n* = 42) or 6 mg/d over 24 weeks	Transdermal placebo patch over 10 weeks (*n* = 43)	128 HIV patients with cognitive impairment (at least 1 SD below the mean in 2 or more neuropsychological tests or 2 SD below the mean in at least one neuropsychological test)	No cognitive improvement observed
Schifitto^51^	2009	Single-arm, open-label study	Lithium, oral application of 300 mg twice daily over 10 weeks (*n* = 13)	None	13 HIV patients with cognitive impairment (at least 1 SD below the mean in 2 or more neuropsychological tests or 2 SD below the mean in at least one neuropsychological test)	No cognitive improvement observed
McElhiney^52^	2010	Randomized, double-blind, placebo-controlled trial	Modafinil, oral application (dose not specified) over 4 weeks (*n* = 59)	Oral placebo over 4 weeks (*n* = 44)	103 HIV patients with fatigue (defined by the Role Function Scale and the Fatigue Severity Scale)	Significant improvement on a composite neuropsychological score restricted to WAIS digit symbol task for processing speed and Grooved Pegboard Test for non-dominant dexterity Significant improvement in subjective cognitive function (Cognitive Failure Questionnaire)
Zhao^53^	2010	Randomized, double-blind, placebo-controlled trial	Memantine (NMDA receptor antagonist), oral application with titration up to 40 mg/d over 20 weeks (*n* = 51)	Oral placebo over 20 weeks (*n* = 48)	99 HIV patients with cognitive impairment (AIDS dementia complex stage 1 or more)	Significant improvement in a global cognitive score (Eight neuropsychological measures NPZ-8)
Simioni^54^	2013	Randomized, double-blind, placebo-controlled crossover trial	Rivastigmin (cholinesterase inhibitor), oral application of up to 12 mg/d for 20 weeks (before or after placebo) (*n* = 17)	Oral placebo over 20 weeks (before or after placebo)	17 HIV patients with cognitive impairment (Frascati criteria)	Improvement in processing speed (Trail Making Test Part A) No improvement in cognitive function (Alzheimer’s Disease Assessment Scale Cognitive Subscale)
Sacktor^55^	2018	Randomized controlled trial with 2 × 2 factorial design	Fluconazole (antimycotic), oral application of 100 mg twice daily for 24 weeks (*n* = 11) Paroxetine (SSRI), oral application of 20 mg/d for 24 weeks (*n* = 11) Fluconazole and paroxetine, 100 mg twice daily and 20 mg/d for 24 weeks (*n* = 12)	Oral placebo over 24 weeks (*n* = 11)	45 HIV patients with cognitive impairment (at least 1 SD below the mean in 3 or more neuropsychological tests or 2 SD below the mean in at least one and 1 SD below the mean in at least one other neuropsychological test)	Significant improvement of patients receiving paroxetine in sequential reaction time (California Computerized Assessment Package), cognitive flexibility [Trail Making Test Part B and verbal fluency (FAS test)] Deterioration of paroxetine groups in multitasking (Letter Number Sequencing) No changes in activities of daily living or performance-based assessments (all groups)

Numerous other pharmacotherapies with a putative anti-inflammatory or neuroprotective mechanism have been studied in people with HIV with NCI. However, none of these yielded significant benefits to cognition.^18^ Still, some pharmacological options with promising results in preclinical studies remain to be explored in the clinical setting. One example is baricitinib, a Janus kinase 1/2 inhibitor, which has been shown to yield a significant reduction in neuroinflammation biomarkers as well as better performance in an object recognition test in a HIV-associated neurocognitive disorder mouse model.^56^

Taken together, the mixed results of studies on cART and the lack of widely recognized adjuvant pharmacological options so far call for developing non-pharmacological approaches such as cognitive neurorehabilitation to address NCI due to HIV.

### Cognitive neurorehabilitation in people with HIV

In what follows, we will review the current evidence on compensatory neuropsychological rehabilitation and restorative technology-assisted approaches such as computerized cognitive training and serious video games. Please refer to Supplementary Table 1 for details.

#### Compensatory neuropsychological rehabilitation for HIV-related NCI

While neuropsychological rehabilitation yields promising outcomes in patients with stroke,^57-59^ multiple sclerosis^60,61^ and mild and moderate dementia,^62,63^ only a few neuropsychological rehabilitation approaches have been studied in individuals with HIV. For instance, a single-arm study investigated a 4-week cognitive neurorehabilitation programme based on compensatory working memory training (spaced retrieval) and the use of external memory aids (e.g. calendars and pill organizers) in 10 elderly people with HIV with executive dysfunction.^64^ The main outcomes consisted of self-set functional aims such as remembering the date, medical appointments or taking medicine. All but one patient achieved their goals, and two-thirds demonstrated some persistence of the learned strategies at a 2-month follow-up. A different single-arm study evaluated the efficacy of Goal Management Training, a group-based cognitive rehabilitation programme, which aimed at educating participants in compensatory strategies such as stress management and self-management principles.^65^ The study included 30 people with HIV with either subjective cognitive deficits or a score below the 50th percentile in a computerized cognitive test performed in a larger study cohort.^20^ Participants underwent a short, computerized neuropsychological assessment and a questionnaire on subjective cognitive function before and after the 9-week intervention with weekly sessions over 2 h. The post-training assessment yielded no significant change in processing speed, attention, memory and executive function compared with pre-training, and no significant difference was found in comparison with a wait-list control group of 23 people with HIV. Out of the 30 participants in the intervention group, nine showed an insufficient adherence to training. In these patients, subjective cognitive function tended to decline, whereas it remained stable in 11 and improved in 10 of the participants adhering to training. In another RCT, 32 people with HIV (16 with and 16 without NCI) were randomly attributed either to a 4-month neuropsychological rehabilitation programme or to standard care.^66^ The cognitive neurorehabilitation consisted of 36 sessions with a set of seven paper-and-pencil exercises mainly targeting compensatory strategies and metacognitive awareness across the domains of attention, bimodal memory and learning, as well as executive function and working memory. A restorative computer-based training for visuospatial attention and memory (COG.I.TO) was also administered. The experimental group improved in learning and memory, executive function, verbal fluency and attention/working memory, whereas the control group exhibited significant decline in those same domains. Noteworthy, benefits of the training transferred to self-reported Instrumental Activities of Daily Living, but most of the gains were lost at 6-month follow-up.

#### Computerized cognitive training

Considering the advent of information and communication technology, computerized cognitive training (CCT) has received increasing interest as an alternative or additional restorative approach to conventional neuropsychological rehabilitation. Indeed, CCT offers the advantages of standardization, reproducibility and flexible training environments—and the opportunity to provide the training doses needed for restoration in an efficient way.^61,63^ In HIV, only few studies have addressed CCT, mainly targeting working memory, processing speed or global cognitive function.^67^

In an RCT, 21 people with HIV with working memory deficits received a 10-week programme of specific verbal and visuospatial working memory CCT using the PSSCogRehab software with either increasing or stable difficulty.^68^ Working memory and sustained attention improved significantly in the group with increasing difficulty as opposed to the group with stable training demands, but benefits were domain specific without transfer to other cognitive domains.

A recent RCT examined the efficacy of the CCT CogniPlus targeting working memory and attention in 60 people with HIV and NCI as per the computerized Vienna Test system, with equal attribution to CCT 90 min twice a week for 12 weeks or a no-contact control group receiving usual care.^69^ The intervention group improved significantly in non-verbal learning, visual memory, planning, spatial, divided and selective attention, as well as global neurocognitive performance, whereas the no-contact group remained stable. These benefits were maintained at long-term assessment 12 weeks after training completion.

In a non-randomized study involving both people with HIV (*n = 30*) and participants without an HIV infection (*n = 30*), a 24-week remote cognitive neurorehabilitation programme (SmartBrain) targeting visual memory and learning, attention and executive functions did not change global cognitive function.^70^ Of note, participants determined the training dose themselves, resulting in a 54% adherence rate, and a highly variable amount of completed activities (from 0 to 941). A dose–response analysis showed a significant improvement of the composite neuropsychological score in the quartile with the highest exposure (at least one 30-min session per week for 24 weeks). Neither group nor dose of training affected subjective cognitive decline and its impact on everyday life as assessed by the Medical Outcomes Survey-HIV. This study further highlights the importance of adherence and training dose for restorative cognitive neurorehabilitation, in a population where adherence issues may well occur.^8^

#### Gamified cognitive neurorehabilitation

Games are known to promote motivation and training engagement.^71,72^ Although commercial video games lack a specific neuroscientific and neuropsychological design, they can be efficacious in achieving cognitive improvement in people with HIV, probably due to challenging several cognitive domains simultaneously. For instance, in an RCT, 11 elderly patients with HIV with NCI trained with a commercial video game (*GT Racing 2*) accompanied by active or sham transcranial direct current stimulation.^73^ After 2 weeks, both the active and sham transcranial direct current stimulation groups improved their scores in verbal learning as well as working memory, with slightly greater although non-significant improvement in the group undergoing active transcranial direct current stimulation.

On the other hand, gamified interventions designed specifically for cognitive training and neurorehabilitation are increasingly considered a promising approach for neurorehabilitation, and restorative gamified CCT has yielded encouraging outcomes in people with HIV.^61,63,71^ In what follows, we will discuss the findings according to the specific cognitive domains targeted by the gamified CCT and highlight potential implications for remote neurorehabilitation.

##### Working memory

In an RCT, 25 sessions of the adaptive and gamified working memory programme Cogmed® were compared with non-adaptive Cogmed® training in people with HIV (*n* = 54) and participants without an HIV infection (*n* = 62), with and without cognitive deficits.^74^ Cogmed® features different working memory tasks that involve the storage of auditory-verbal or visuospatial information, with or without manipulation of the information. Irrespective of serological status, the adaptive training group showed significantly improved performance in working memory compared with non-adaptive training. There was no distal transfer to untrained cognitive domains. Moreover, fMRI analyses from participants with HIV in the adaptive training group (*n = 19*) showed decreased brain activation in the right middle frontal gyrus that correlated with greater scores on one measure of verbal working memory (backward digit span), interpreted as improved neural efficiency. The authors also analysed the polymorphism of the LIM homeobox transcription factor-1-alpha gene (LMX1A-rs4657412). This gene polymorphism is believed to reflect greater dopaminergic reserve, potentially predisposing to greater working memory training benefits.^75^ Indeed, greater improvements and persistence of working memory benefits at the 6-month follow-up assessment were seen for patients with an LMX1A genotype compared with non-carriers, especially in the HIV group.

##### Processing speed

Forty-six people with HIV were assigned randomly to either the Posit Science InSight CCT targeting visuospatial processing and memory or a no-contact control group.^76^ Compared with the no-contact group, the 10-h gamified training afforded significant improvements in visuospatial processing speed as well as sustained and divided attention. These effects were assessed by the useful field of view test, which has been reported to be associated with driving ability in patients with traumatic brain injury.^77,78^ More importantly, the trained group also showed significant improvements in a visuospatial processing speed test that is hypothesized to relate to everyday functioning (Timed Instrumental Activities of Daily Living).^79^ The vast majority of the interventional group reported cognitive improvement.

Similar results were afforded by a home-based training protocol within a single-arm study in 20 adults with HIV playing the Posit Science Road Tour game (part of Posit Science BrainHQ) targeting processing speed.^80^ Processing speed and everyday functioning improved significantly after 10 h of training. Moreover, the majority of participants indicated game enjoyment at a moderate to high level.

A case comparison study reported improved processing speed for two people with HIV and NCI training with Posit Science BrainHQ over 10 and 20 h, respectively, as compared with the one patient engaged in sham internet-based activity as an active control.^81^ Only the patient undergoing 20 h of gamified CCT did no longer meet the Frascati criteria.

However, a recently published three-arm RCT on the processing speed module of Posit Science BrainHQ in 216 people with HIV and NCI (according to Frascati criteria) did not yield any significant benefits as compared with an active control group.^82^ Here, participants were randomized to either 20 h (*n* = 73) or 10 h (*n* = 70) of CCT, or to a control group (*n* = 73) engaging in 10 h of unspecific ‘Internet Navigation Control Training’ comprising a number of health-related and general web-based activities. Sensitivity analyses suggested post-training improvements in processing speed and global cognition of the interventional over the control groups, considered irrelevant by the authors.

##### Multidomain gamified cognitive training

Multidomain training with Posit Science BrainHQ was assessed in two RCTs. Twenty-four people with HIV and a Montreal Cognitive Assessment score < 26 underwent home-based training with Posit Science BrainHQ or received weekly health-related newsletters and follow-up calls over 8 weeks.^83^ After the 8-week training period, the interventional group exhibited a significant, yet small (effect size partial *η*^2^ 0.32) improvement on the Montreal Cognitive Assessment score compared with the control group. This benefit persisted at 8- and 16-week follow-ups. However, more detailed neuropsychological evaluation and assessment of everyday cognition were not performed in this study.

In another RCT on Posit Science BrainHQ, 48 people with HIV-associated neurocognitive disorder engaged in 12 weeks of CCT, while 40 participants were attributed to a no-contact control group. Patients in the intervention group performed 20 h of CCT on two of their deficient cognitive domains.^84^ In case of more than two impaired domains, the authors chose to train the least impaired domains in which improvement would most likely result in reversal of the Frascati criteria. Processing speed training (*n* = 22) showed significant and moderate to strong effects on processing speed, attention, verbal learning and memory, delayed verbal memory and executive functioning, as compared with the control group. However, only three participants (6.3%) of the interventional group no longer met the Frascati criteria, compared with 6 (15.0%) in the no-contact control group.

##### Potential impact at the global scale

An additional advantage of digital gamified neurorehabilitation approaches for cognitive deficits due to HIV is their potential worldwide availability. For example, an RCT in South African HIV-positive adolescents between the ages of 10 and 16 years used the adaptive serious video game Jungle Memory with three modules (first targeting verbal memory, word recognition and processing speed; second targeting visuospatial working memory; third targeting numerical reasoning and sequential memory).^85^ In this study, 31 participants were assigned to the intervention group, and 32 participants engaged in non-specific computerized control training. Training duration was 8 weeks, with 32 supervised, half-hour sessions in both groups. Compared with the control group, the intervention group improved significantly in several tests of verbal working memory. This improvement persisted up to the long-term evaluation 6-month post-training. Interestingly, Jungle Memory participants also improved in measures of attention, memory, executive function, language and fluid intelligence.

An RCT in 60 school-age Ugandan children living with HIV compared a no-contact group to Captain’s Log, a 10-session gamified CCT protocol targeting executive function, visuomotor skills, attention and memory.^86^ The authors reported significant improvements on visuomotor executive function, learning and processing speed in the active group. The 95% adherence rate underlines the feasibility and acceptability of gamified CCT and the ease of use of such interventions.

Implementing a similar design, a larger three-arm RCT on Captain’s Log was conducted in 159 Ugandan children with HIV.^87^ Children with HIV were either randomized to a no-contact control group (*n* = 54) or to one of two intervention groups receiving 24 hourly sessions of Captain’s Log with adaptive (*n* = 53) or randomly alternating difficulty levels (*n* = 52). All but two participants completed the study (attrition rate 1.3%). Both CCT groups improved significantly and sustainably in processing speed and in visuomotor executive function and learning, as compared with the passive control group. Furthermore, children in the adaptive CCT group showed specific significant improvements (compared with the control group) in overall performance on global cognitive function, as well as crystalline intelligence and planning, at the post-training and 3-month follow-up. The non-adaptive CCT group performed better in learning compared with the control group after the training and at the follow-up assessment, whereas the adaptive CCT group only showed a trend for improved learning.

Captain’s Log was also assessed in older Ugandan individuals.^88^ Like the studies performed in children, this non-randomized study in 81 older individuals in Uganda (including 40 people with HIV) showed a high completion rate of more than 90%. Moreover, the authors reported promising effect sizes, albeit not statistically significant, for immediate and delayed verbal memory tests, verbal working memory, verbal fluency, cognitive flexibility and fine motor function in the 21 people with HIV training with Captain’s Log, compared with participants in the no-contact group.

## Discussion

Affecting around 4 million people worldwide, NCI among people with HIV represents a major concern, interfering with everyday socio-professional activity, quality of life, medication adherence and potentially contributing to mortality.^6-10^ Consequently, there is a strong need to optimize both diagnosis and treatment of NCI among people with HIV. This narrative review encompasses a recommendation for the interdisciplinary assessment of NCI based on recent guidelines and expert consensus^13^ and provides an overview of different pharmacological and neurorehabilitation treatment options under investigation.

To this date, screening for NCI in people with HIV only has a marginal role. As mentioned above, both the Mini Mental State Examination and Montreal Cognitive Assessment have been examined in this context but demonstrate a low sensitivity for detecting NCI in this population.^23,24^ Establishing effective cognitive screening tools may prove beneficial for the care of people with HIV, especially in low- and middle-income countries and rural areas with limited access to a comprehensive work-up. With the potential for automated and remote testing independent of skilled personnel, the development of digital screening tools may have a decisive advantage over conventional screening methods and may be worth pursuing by future research. Of note, digital screening initiatives have been launched for multiple sclerosis and NCI.^89,90^

Meanwhile, diagnosis and classification of NCI in people with HIV still primarily rely on neuropsychological testing and the presence of cognitive complaints. In the differential diagnosis, two major factors have to be considered. First, NCI among people with HIV is assumed to be of multifactorial origin, ranging from HABI itself (e.g. CNS viral escape) to cART-related neurotoxicity.^18^ Second, people with HIV have a higher prevalence of co-morbidities affecting cognition, such as anxiety and depression, substance use or opportunistic CNS infections.^19,91-94^ Consequently, in patients with significant cognitive complaints interfering with everyday life,^25^ a structured and interdisciplinary diagnostic work-up is recommended, consisting of infectious disease, neurological, neuropsychological and psychiatric evaluations, allowing for better management of the contributing co-morbidities (such as mood disorders or substance use).^21^

With respect to treatment of HABI, there is a lack of a gold standard, guidelines or even widely used, efficient approaches. While the widespread use of cART proved effective for decelerating cognitive decline^16^ and reducing the prevalence of HIV-associated dementia,^11^ cART alone is unable to alleviate the burden of NCI among people with HIV. Interestingly, the effects of administering cART regiments with a higher CPE remain controversial.^40-42^ One possible explanation is that medication with a higher CPE may prevent damage resulting from CNS viral escape syndrome but simultaneously yield more drug-related CNS toxicity.^43^ As an alternative explanation, HIV may provoke persisting compartmentalized inflammation that seems refractory to cART.^95,96^

Numerous studies explored the potential of adjuvant pharmacological treatment options (Table 2). Modafinil represents one of the more promising agents, with one study demonstrating improvement on a global cognitive score, fatigue and subjective cognitive function, even if cognitive performance of the patients improved in only two domains.^52^ However, the evidence remains low, including the majority of anti-inflammatory or neuroprotective agents, partly due to limited sample sizes^48,49,51,54^ or mixed results.^50,53,55^

While the evidence for pharmacological options remains limited, cognitive neurorehabilitation appears to represent a promising avenue for the management of HIV-related NCI. One study showed that predominantly compensatory conventional neuropsychological rehabilitation may lead to improvements—if only transient—in both targeted domains and activities of daily living.^66^ Results of a different study suggest that compensatory neuropsychological rehabilitation seems to increase the ability to achieve self-set functional goals in most patients, with a sustained effect in a majority.^64^ A study with a similar compensatory approach reported improved subjective cognitive function in 70% of the patients adhering to the treatment, even if no change in objective cognitive function was noted.^65^ Due to the limited sample sizes, however, these results have to be interpreted with caution. Well-designed, large-scale RCTs are needed to provide sufficient evidence for specific cognitive neurorehabilitation approaches for NCI among people with HIV.

Future cognitive neurorehabilitation programmes could benefit from integrating multimodal restorative technology-assisted interventions with compensatory and metacognition-related approaches, such as in RCTs underway for early dementia.^97^ Technology-assisted approaches may offer important advantages over conventional restorative approaches. For instance, digital therapeutics enable high levels of both standardization and personalization, as well as improved accessibility and potentially higher rehabilitation dose, given lower needs in specialized supervision for restoration.^61,63^ In addition, technology may facilitate the extension of cognitive neurorehabilitation to the home setting that may prove of fundamental value for patients with limited access to the healthcare system.^86,98^

In particular, gamified CCT and serious video games appear promising for cognitive neurorehabilitation. Given the immersive graphics and varied environments, gamified CCT and serious video games are usually perceived as highly entertaining and engaging, including people with HIV.^86^ The sense of enjoyment and motivation leads to excellent adherence.^86-88^ Maintaining adequate levels of motivation appears to represent a decisive factor for successful cognitive neurorehabilitation,^61,63^ especially taking into account the high prevalence of apathy and depression in people with HIV.^99^

Interestingly, even commercial video games seem to improve the cognitive domains of attention, processing speed and cognitive flexibility.^72^ However, these games lack thorough neuroscientific and neuropsychological development and validation.^71^ Such a design can allow integrating both the motivational aspects of video games and neuroscientifically valid multimodal contents needed for efficacious restoration and impact on everyday cognition and life.^61,63^ Neuroscience-inspired adaptive serious video games have indeed afforded promising cognitive outcomes in healthy older people^100,101^ and adolescents with attention deficit and hyperactivity disorder.^102^

Several RCTs have evaluated gamified cognitive neurorehabilitation in people with HIV. Among the most studied is the multidomain gamified CCT Posit Science BrainHQ. Although varying in study methodology—including the selection of subtasks—significant improvement has been reported in processing speed, one of the most frequently impaired domains among people with HIV,^76,80,82-84^ with several studies suggesting potential relevance for processing speed in everyday life activities.^76,83,84^ Other notable examples of gamified CCT and video games studied in NCI among people with HIV include Cogmed (various bimodal working memory tasks) and Captain’s Log (multimodal CCT targeting executive function, visuomotor skills, attention and memory), with some evidence pointing towards improved verbal working memory after training with the former^74^ and an increase in visuomotor executive function and processing speed after training with the latter.^86,87^ A different study in adolescents with HIV using the serious video game Jungle Memory with three adaptive modules (first targeting verbal memory, word recognition and processing speed; second targeting visuospatial working memory; and third targeting numerical reasoning and sequential memory) demonstrated long-term improvements in verbal (but not visuospatial) working memory and distant transfer to untrained cognitive domains.^85^ Taken together, gamified cognitive neurorehabilitation of people living with HIV remains a promising option but still lacks convincing results. Furthermore, upon sufficient evidence in favour of such digital therapeutics, given the need for specific interventions adapted to the patient’s profile, their use should be prescribed and closely monitored by skilled clinicians acquainted with their content.

On the other hand, despite these encouraging results, truly compelling evidence for specific approaches is still lacking, likely due to methodological shortcomings. Most importantly, the majority of data in the field stems from relatively constrained sample sizes. Additionally, only six of the 18 reviewed cognitive neurorehabilitation studies reported effect sizes of their interventions,^69,73,82-84,88^ whereas effect sizes are crucial for interpretation and meta-analyses of the data.

Several other major factors have to be considered when designing future trials on cognitive neurorehabilitation in NCI among people with HIV (Table 3). As already mentioned above, one of the more pertinent issues concerns the selection of adequate inclusion criteria. Only five of the 18 reviewed studies on cognitive neurorehabilitation specifically included people with NCI according to the Frascati criteria^73,81,82,84^ or based on similar cut-off values.^69^ Several studies included people with HIV regardless of cognitive function, bearing the risk for high heterogeneity of the data. The baseline level of cognitive impairment may also determine the choice of the neurorehabilitation approach and influence the outcomes. Applying relatively strict inclusion criteria based on cognitive complaints by affected individuals—or report of their proxies—together with cut-off values in neuropsychological tests in frequently affected domains (processing speed, attention, working memory and cognitive flexibility) and covariate-adaptive randomization may be helpful in designing future RCTs.

**Table 3:** Methodological considerations for future cognitive neurorehabilitation studies for NCI in people with HIV

Study design	Randomized controlled trial Participant number based on power analysis Expectancy-matched, active control condition Blinded assessors
Eligibility criteria	Diagnosis of NCI: At least 1 SD below the mean in at least 2 out of seven tested cognitive domains (for recommendations, see Table 1)
Outcome measures	Patient-reported outcome measures (at least quality of life, subjective cognitive function, activities of daily living; consider also behavioural and psychiatric co-morbidities) Neuropsychological tests on several cognitive domains (at least processing speed, attention, working memory and cognitive flexibility as the most common; for recommendations, see Table 1) Long-term follow-up testing (at least 3 months after conclusion of the intervention)
Statistics	Mixed-linear models for data analysis Calculation and reporting of effect sizes

Suggestions based on limitations of previous studies.

Moreover, careful selection of the outcome measures is key for obtaining clinically relevant results. Measuring changes in at least the most frequently impaired domains in HIV may yield a comprehensive overview about the effect of an intervention, including assessments on transfer to untrained domains. The ultimate goal of neurorehabilitation is sustainable improvement, thus measuring the persistence of potential cognitive benefits over time appear essential, but has only been implemented in six of the 18 reviewed studies.^69,74,82,83,87,91^ Furthermore, measures of activity and participation need to be developed and implemented, in order to assess the impact on everyday life, also at follow-up.

Careful consideration is warranted in terms of the selected control condition. Almost half of the reviewed RCTs on cognitive neurorehabilitation included a no-contact control condition. However, this is equivalent to omitting a placebo drug in a pharmacological study—participants are aware of not receiving any intervention. The absence of an active control condition (including passive movie watching) can be compared with unveiling the placebo drug, with participants’ lower expectations contributing to differences in behavioural outcomes.^103,104^ Even if designing an active control intervention is challenging in cognitive neurorehabilitation, state-of-the-art RCTs should include expectancy-matched cognitive interventions with low, different or unspecific effects to avoid significant differences in expected and thus resulting benefits.^61,63,103^

Potential cognitive benefits of physical exercise in people with HIV remain an open question. In contrast to healthy individuals,^105^ thus far, no conclusive data are available supporting physical exercise for cognitive improvement in people with HIV. However, the outcomes of a large-scale RCT comparing 6 months of aerobic physical exercise training to stretching in elderly people with HIV are expected soon,^106^ with the potential of paving the way for combined physical and cognitive exercise in cognitive neurorehabilitation, such as currently explored in multiple sclerosis and NCI.^61,63,101,107^

Finally, the optimal dose, frequency and session duration of cognitive neurorehabilitation for NCI among people with HIV remain unclear, although some evidence suggests dose–response relationships in CCT.^69^ In the reviewed studies, training frequency in gamified CCT—where specified—varied between slightly over once per week^68^ to four times a week.^88^ Evidence on cognitive training in healthy older adults suggests a minimal training session duration of 30 min, a training frequency between once and three times a week and a training dose of at least 20 h.^108^ Studies on cognitive neurorehabilitation in patients with multiple sclerosis adopted comparable training dose and frequency.^61,109^ However, dose–response relationships remain unclear and have been little investigated in people with HIV-related NCI. Future research to establish the optimal training dose and frequency in this population is necessary, in particular as studies in other populations such as older adults at risk for neurocognitive decline suggest a non-linear dose–response function,^110^ and these relationships may vary depending on the underlying pathology.

## Conclusion

NCI is a frequent and detrimental condition in millions of people with HIV worldwide. With multiple factors contributing, a multidisciplinary panel of specialists in infectious disease, neurology, neuropsychology and psychiatry appears indispensable for assessment and management in case of everyday-relevant cognitive impairment. Treatment options for NCI among people with HIV remain limited, although some pharmacological and non-pharmacological interventions have yielded promising results. Future large-scale cognitive neurorehabilitation RCTs using state-of-the-art methodology in terms of inclusion criteria, expectancy-matched active control conditions, comprehensive cognitive outcome measures, assessments of effects on everyday life and long-term outcomes are needed to alleviate the burden of NCI among people with HIV. Taken together, a refined interdisciplinary assessment and multimodal cognitive neurorehabilitation including neurotechnological approaches for restoration will pave the way towards optimal care and treatment options for an often neglected yet substantial issue affecting the personal and professional well-being of people with HIV.

## Supplementary Material

fcae399_Supplementary_Data

## Data Availability

Data sharing is not applicable to this article as no new data were created or analysed in this study.
