# Supplementary material for: Advances in assessment and cognitive neurorehabilitation of HIV-related neurocognitive impairment
Source: Brain Commun. 2024 Dec 26;7(1):fcae399. doi: 10.1093/braincomms/fcae399 (PMC11670355; doi:10.1093/braincomms/fcae399)
Supplement: fcae399_Supplementary_Data [file fcae399_supplementary_data.docx]

**Advances in assessment and cognitive neurorehabilitation of HIV-related neurocognitive impairment**

**Supplementary Online Material**

**Supplementary Table 1: A summary of studies on cognitive neurorehabilitation in NCI among people with HIV**

| **First Author,**  **Year** | **Cognitive domain trained** | **Study Arms** | **Population** | **Outcome measures** | **Transfer on far objective cognitive outcomes** | **Transfer on everyday life** |
| --- | --- | --- | --- | --- | --- | --- |
| **Neundorfer,**  **2004**^1^ | Compensatory strategy | Working memory training and use of external memory aids (n=10)  Control: None | 10 people with HIV (≥50 y.o.) with executive functioning impairment | 9/10 patients achieved their goals, and majority had stability on the learned strategy at two months | N/A | N/A |
| **Boivin,**  **2010**^2^ | Attention, memory and learning, executive functioning and visual motor skills | 10 sessions (over 5 weeks) of Captain's Log computer program (n=32)  Control: No-contact group (n=28) | 60 children with HIV infection | Improved executive function and visuo-motor skill (Cogstate assessment battery) but no difference in memory | Not examined | Not examined |
| **Becker,**  **2012**^3^ | Memory, attention, executive function, gnosis | 24 weeks of SmartBrain © Cognitive stimulation program, at home with variable degree of use (n=46)  Control: No-contact group (n=14) | 60 patients without confirmed cognitive disorder (whereof 30 HIV-positive) | No main effect on global cognitive function (GIR) but significant dose-response relationship of training and global cognitive function (GIR) | Not examined | No effect on quality of life (MOS-HIV) |
| **Vance,**  **2012**^4^ | Processing speed | 10 hours of the PositScience InSight computer program (n=22)  Control: No-contact group (n=24) | 46 adults with HIV and no defined cognitive impairment | Improved processing speed(UFOV and TIADL respectively) compared to control group | No transfer effect on executive function (WCST) and fine motor function (finger tapping test) | Improved everyday functioning (TIADL) compared to control group reported |
| **Cody,**  **2015**^5^ | Processing speed | 10 hours (over 5 weeks) of remote PositScience InSight computer program (n=20)  Control: None | 20 adults with HIV (≥40 y.o.) and no defined cognitive impairment | Improved processing speed and everyday functioning (UFOV and TIADL, respectively) | No transfer effect on executive function (WCST) and fine motor function (finger tapping test) | Improved everyday functioning (TIADL) reported |
| **Livelli,**  **2015**^6^ | Attention, visual-verbal working memory, executive functioning and metacognitive awareness | 36 sessions of 8 pencil-and-paper and computer exercises (COG.I.TO.) during 4 months (n=16)  Control: No-contact group (n=16) | 32 people with HIV (16 with and 16 without HAND) | Improved learning and memory (RAVLT-IR, RAVLT-DR, ROCF-DR), executive functioning (ToL, Stroop Errors, TMT-B, FAB, ROCF-C), verbal fluency (FAS, VS), attention/working memory (via extensive assessment) as well as daily functioning (IADL) compared to control group | Not examined | Not examined |
| **Ownby,**  **2016**^7^ | Unspecified | 6 sessions of commercial video game training (GT Racing 2) and active tDCS over 2 weeks (n=6)  Control: Same intervention combined with sham tDCS (n=5) | 11 people with HIV with MND (according to Frascati criteria) | Both groups improved in verbal learning (HVLT-R) and working memory (forward and backward digit span) on neuropsychological assessments, non-significant greater improvement in tDCS group | N/A | Not examined |
| **Boivin,**  **2016**^8^ | Attention, memory and learning, executive functioning and visual motor skills | 24 one-hour sessions (over 2 months, three times a week) of Captain's Log computer program; training in first study arm with adaptive difficulty (n=53), training in second study arm with randomly varying difficulty (n=52)  Control: No-contact group (n=54) | 159 children with HIV infection | Results for post-training assessment and three-month follow-up:  First intervention group with global cognitive improvements (KABC-II) and planning (KABC-II subtest)  Second intervention group improved in learning (KABC-II subtests)  Both intervention groups with improved visuo-motor processing speed , visuo-motor executive function, processing speed (Cogstate assessment battery) | First intervention group with improvements in crystalline intelligence (KABC-II subtest) | Not examined |
| **Chang,**  **2017**^9^ | Working memory | 20 - 25 sessions (over 5 - 8 weeks) of working memory training using CogMed (n=76)  Control: Same inactive training modules, without increasing difficulty (n=40) | 54 people with HIV and 62 non-HIV participants | Improved attention/working memory and executive function (Digit Span forward/backward test, and Spatial Span forward/backward test) compared to control condition | Not examined | Not examined |
| **Hossain,**  **2017**^10^ | Processing speed | 20 hours (A) or 10 hours (B) of PositScience BrainHQ computer program (n=3)  Control: 10 hours of sham internet training (C) | 3 people with HIV with HAND (according to Frascati criteria) | Improved processing speed (UFOV) for A and B; no longer HAND diagnosis for A | Not examined | Not examined |
| **Towe,**  **2017**^11^ | Verbal and visuo-spatial working memory | 12 sessions (over 10 weeks) of a computer-based verbal and visuo-spatial working memory training (PSSCogRehab software) (n=11)  Control: Same inactive training modules, without increasing difficulty (n=10) | 21 people with HIV with working memory impairment | Improved working memory (PASAT-50 and NAB Digit Span) compared to control group | Not examined | Not examined |
| **Frain,**  **2018**^12^ | General cognitive function | 24 sessions (over 8 weeks) of home-based PositScience BrainHQ computer program (n=12)  Control: Weekly health-related newsletter via email and follow-up phone calls (n=12) | 24 people with HIVwith MCI | Improved overall cognitive function (MoCA) compared to control group persisting at 8-week and 16-week follow-up | Not examined | Not examined |
| **Fraser,**  **2020**^13^ | Working memory | 32 half-hour sessions (over 8 weeks) of Jungle Memory (supervised adaptive serious video game training) (n=31)  Control: 32 half-hour sessions (over 8 weeks) of supervised, unspecific training (Microsoft paint) (n=32) | 63 adolescents with HIV between the ages of 10-16 years | Improved verbal but not visuospatial working memory (AMWA) with stable effects up to 6 months after training | Transfer training effects on attention, memory, executive function, language and fluid intelligence (All NEPSY-II) | Not examined |
| **Ezeamama,**  **2020**^14^ | Attention, memory and learning, executive functioning and visual motor skills | At least 20 sessions (over 5 weeks) of Captain’s Log computer program (n=41)  Control: No-contact group (n=40) | 40 people with HIV and 41 non-HIV participants | Improved immediate and delayed recall (AVLT), working memory (backward digit span), verbal fluency (number of animals named in 60s), executive function (CTT) and fine motor function (finger tapping test) | Not examined | Not examined |
| **Vance,**  **2021**^15^ | Individual (two least impaired cognitive domains) | 20 hours (over 12 weeks on average) of PositScience BrainHQ (n=48)  Control: No-contact group (n=40) | 88 people with HIV with HAND diagnosis (according to Frascati Criteria) | Significant mild to moderate post-training effects:  Processing speed training (N=22): Processing speed (Stroop, TMT-A) | Significant mild to moderate post-training effects:  Processing speed training (N=22): Attention (PASAT), Verbal learning (HVLT-R), Delayed verbal memory (HVLT-R), Executive functioning (Stroop Interference, TMT-B)  Attention training (N=16): Delayed verbal memory (HVLT-R), Executive functioning (Stroop Interference, TMT-B)  Delayed verbal memory training (N=13): Processing speed (Stroop, TMT-A), Executive functioning (Stroop Interference, TMT-B)  Executive function training (N=4): Processing speed (Stroop, TMT-A), Attention (PASAT)  Spatial learning and memory training (N=8): Processing speed (Stroop, TMT-A)  Delayed spatial memory training (N=8): Attention (PASAT) | Not examined |
| **Etesami,**  **2022**^16^ | Working memory, visual memory, different domains of attention, nonverbal learning | 24 sessions of 90 minutes (over 12 weeks) of CogniPlus (n=30)  Control: No-contact group (n=30) | 60 people with HIV with NCI (determined by the VTS) | Significant improvement in nonverbal learning, visual memory, divided attention, visuospatial attention and selective attention (all tested with the VTS) in intervention group post-training versus pre-training, stable at the 12-week follow-up | Significant improvement in planning | Not examined |
| **Mayo,**  **2022**^17^ | Compensatory strategy | 9 sessions of 120 minutes (over 9 weeks) of Goal Management Training (n=30)  Control: No-contact group (n=23) | 30 people with HIV with subjective cognitive complaints or score below the mean of a computerized testing (intervention group) and 23 HIV positive patients (control group) | No effect on objective cognitive function (processing speed, attention, memory, executive function, B-CAM) in either group | N/A | Improved or stable subjective cognitive function (C3Q) in 21 patients in the intervention group adhering to training  9 non-adherent patients in the intervention group with tendency towards deterioration of subjective cognitive function (C3Q) |
| **Vance,**  **2024**^18^ | Processing speed | 10 hours (n=70) or 20 hours (n=73) of PositScience BrainHQ (Processing speed modules)  Control: 10 hours of unspecific Internet Navigation Control Training | 216 people with HIV with HAND or borderline HAND (according to frascati criteria) | Significant improvement in processing speed in both intervention groups compared to control group after training but none observed at the 1 year or 2 year follow-up | Significant improvement in global cognitive function in both intervention groups compared to control group after training but none observed at the 1 year of 2 year follow-up  No improvements in other cognitive domains | Not examined |

Abbreviations (alphabetical order):
AMWA: The Automated Working Memory Assessment; AVLT: WHO-UCLA Auditory Verbal Learning Test; B-CAM: brief computerized cognitive assessment battery; CTT: Color Trails Test; C3Q: Communicating Cognitive Concerns Questionnaire; FAS: Phonematic Fluency Test; HAND: HIV-Associated Neurocognitive Disorder; HVLT-R: Hopkins Verbal Learning Test-Revised; GIR: Globar Impairment Rating composite score of neuropsychological tests; KABC-II: Kaufmann Assessment Battery for Children second edition; MND: Mild Neurocognitive Disorder; MOS-HIV: Medical Outcomes Study HIV Health Survey; NAB: Neuropsychological Assessment Battery; NEPSY-II: A Developmental Neuropsychological Assessment – Second Edition; PASAT: Paced Auditory Serial Addition Test; RAVLT-DR: Rey Auditory Verbal Learning Test Delayed Recall; RAVLT-IR: Rey Auditory Verbal Learning Test Immediate Recall; ROCF-C: Rey-Osterrieth Complex Figure Copy; ROCF-DR: Rey-Osterrieth Complex Figure Copy; Stroop: Stroop Color Test; (T)IADL: (Timed) Instrumental Activities of Daily Living; tDCS: transcranial Direct Current Stimulation; TMT-A: Trail Making Test Part A; TMT-B: Trail Making Test Part B; ToL: Tower of London; UFOV: Useful Field of View Test; VS: Verbal Span; VTS: Vienna Test System Package; WCST: Wisconsin Card Sorting Test.

**Supplementary References**

1. Neundorfer MM, Camp CJ, Lee MM, Skrajner MJ, Malone ML, Carr JR. Compensating for Cognitive Deficits in Persons Aged 50 and Over with HIV/AIDS. *Journal of HIV/AIDS & Social Services*. 2004;3(1):79-97. doi:10.1300/J187v03n01_07
2. Boivin MJ, Busman RA, Parikh SM, et al. A pilot study of the neuropsychological benefits of computerized cognitive rehabilitation in Ugandan children with HIV. *Neuropsychology*. Sep 2010;24(5):667-73. doi:10.1037/a0019312
3. Becker JT, Dew MA, Aizenstein HJ, et al. A pilot study of the effects of internet-based cognitive stimulation on neuropsychological function in HIV disease. *Disabil Rehabil*. 2012;34(21):1848-52. doi:10.3109/09638288.2012.667188
4. Vance DE, Fazeli PL, Ross LA, Wadley VG, Ball KK. Speed of processing training with middle-age and older adults with HIV: a pilot study. *J Assoc Nurses AIDS Care*. Nov-Dec 2012;23(6):500-10. doi:10.1016/j.jana.2012.01.005
5. Cody SL, Fazeli PL, Vance DE. Feasibility of a Home-Based Speed of Processing Training Program in Middle-Aged and Older Adults With HIV. *J Neurosci Nurs*. Aug 2015;47(4):247-54. doi:10.1097/JNN.0000000000000147
6. Livelli A, Orofino GC, Calcagno A, et al. Evaluation of a Cognitive Rehabilitation Protocol in HIV Patients with Associated Neurocognitive Disorders: Efficacy and Stability Over Time. *Front Behav Neurosci*. 2015;9:306. doi:10.3389/fnbeh.2015.00306
7. Ownby RL, Acevedo A. A pilot study of cognitive training with and without transcranial direct current stimulation to improve cognition in older persons with HIV-related cognitive impairment. *Neuropsychiatr Dis Treat*. 2016;12:2745-2754. doi:10.2147/NDT.S120282
8. Boivin MJ, Nakasujja N, Sikorskii A, Opoka RO, Giordani B. A Randomized Controlled Trial to Evaluate if Computerized Cognitive Rehabilitation Improves Neurocognition in Ugandan Children with HIV. *AIDS Res Hum Retroviruses*. Aug 2016;32(8):743-55. doi:10.1089/AID.2016.0026
9. Chang L, Lohaugen GC, Andres T, et al. Adaptive working memory training improved brain function in human immunodeficiency virus-seropositive patients. *Ann Neurol*. Jan 2017;81(1):17-34. doi:10.1002/ana.24805
10. Hossain S, Fazeli PL, Tende F, Bradley B, McKie P, Vance DE. The Potential of Computerized Cognitive Training on HIV-Associated Neurocognitive Disorder: A Case Comparison Study. *J Assoc Nurses AIDS Care*. Nov - Dec 2017;28(6):971-976. doi:10.1016/j.jana.2017.06.011
11. Towe SL, Patel P, Meade CS. The Acceptability and Potential Utility of Cognitive Training to Improve Working Memory in Persons Living With HIV: A Preliminary Randomized Trial. *J Assoc Nurses AIDS Care*. Jul - Aug 2017;28(4):633-643. doi:10.1016/j.jana.2017.03.007
12. Frain JA, Chen L. Examining the effectiveness of a cognitive intervention to improve cognitive function in a population of older adults living with HIV: a pilot study. *Ther Adv Infect Dis*. Jan 2018;5(1):19-28. doi:10.1177/2049936117736456
13. Fraser S, Cockcroft K. Working with memory: Computerized, adaptive working memory training for adolescents living with HIV. *Child Neuropsychol*. Jul 2020;26(5):612-634. doi:10.1080/09297049.2019.1676407
14. Ezeamama AE, Sikorskii A, Sankar PR, et al. Computerized Cognitive Rehabilitation Training for Ugandan Seniors Living with HIV: A Validation Study. *J Clin Med*. Jul 7 2020;9(7)doi:10.3390/jcm9072137
15. Vance DE, Fazeli PL, Azuero A, Wadley VG, Raper JL, Ball KK. Can Individualized-Targeted Computerized Cognitive Training Benefit Adults with HIV-Associated Neurocognitive Disorder? The Training on Purpose Study (TOPS). *AIDS Behav*. Dec 2021;25(12):3898-3908. doi:10.1007/s10461-021-03230-y
16. Etesami MS, Saboury N, Mohraz M, et al. Immediate and Long-Term Effects of a Computerized Cognitive Rehabilitation Therapy on Cognitive Function in People Living with HIV in Iran: A Single-Blind Two-Arm Parallel Randomized Controlled Trial. *J Assoc Nurses AIDS Care*. Sep-Oct 01 2022;33(5):505-522. doi:10.1097/JNC.0000000000000339
17. Mayo NE, Levine B, Brouillette MJ, Belanger D, Fellows LK. Efficacy potential of Goal Management Training to improve cognitive function in older people living with HIV. *Contemp Clin Trials Commun*. Dec 2022;30:101023. doi:10.1016/j.conctc.2022.101023
18. Vance DE, Fazeli PL, Azuero A, et al. A 2-year longitudinal randomized controlled trial examining the transfer of speed of processing training to secondary cognitive domains in middle-aged and older adults with HIV-associated neurocognitive disorder: Results of the think fast study. *Clin Neuropsychol*. Feb 2024;38(2):471-492. doi:10.1080/13854046.2023.2212867
